# Exploratory analysis of genomic segmentations with Segtools

**DOI:** 10.1186/1471-2105-12-415

**Published:** 2011-10-26

**Authors:** Orion J Buske, Michael M Hoffman, Nadia Ponts, Karine G Le Roch, William Stafford Noble

**Affiliations:** 1 Department of Genome Sciences, University of Washington, PO Box 355065, Seattle, WA 98195-5065, USA; 2 The Institute for Integrative Genome Biology, University of California, Riverside, 900 University Avenue, Riverside, CA 92521, USA; 3 Department of Computer Science and Engineering, University of Washington, PO Box 352350, Seattle, WA 98195-2350, USA

## Abstract

**Background:**

As genome-wide experiments and annotations become more prevalent, researchers increasingly require tools to help interpret data at this scale. Many functional genomics experiments involve partitioning the genome into labeled segments, such that segments sharing the same label exhibit one or more biochemical or functional traits. For example, a collection of ChlP-seq experiments yields a compendium of peaks, each labeled with one or more associated DNA-binding proteins. Similarly, manually or automatically generated annotations of functional genomic elements, including *cis*-regulatory modules and protein-coding or RNA genes, can also be summarized as genomic segmentations.

**Results:**

We present a software toolkit called *Segtools *that simplifies and automates the exploration of genomic segmentations. The software operates as a series of interacting tools, each of which provides one mode of summarization. These various tools can be pipelined and summarized in a single HTML page. We describe the Segtools toolkit and demonstrate its use in interpreting a collection of human histone modification data sets and *Plasmodium falciparum *local chromatin structure data sets.

**Conclusions:**

Segtools provides a convenient, powerful means of interpreting a genomic segmentation.

## Background

Genomic research often requires classifying regions of the genome according to their biochemical or functional properties and then investigating how these classes relate to one another and to complementary genomic data sets. One might create these classifications automatically, by using machine learning methods that partition the genome into labeled segments [1-4], or manually on the basis of one or more experimental data sets.

In either case, one then faces the challenge of exploring the biological meanings of the segment labels. The UCSC Genome Browser [5] allows researchers to explore some such relationships manually, but such analyses do not efficiently scale to aggregation over a complete genome. The Galaxy platform [6] and BEDTools [7] provide useful large-scale automated analyses, but these methods do not generate the aggregate comparisons and visualizations critical to understanding these genomic segmentations. EpiGRAPH [8] is a more sophisticated software toolkit that, in conjunction with Galaxy, offers some visualization capabilities in addition to a variety of machine learning analysis methods.

To address this type of analytical challenge, we have developed *Segtools*, a software toolkit that facilitates the exploratory analysis of genomic segmentations. Segtools is designed to provide segmentation-centric summary statistics and visualizations, in a manner that is scalable and easy to use. In this context, a *segmentation *is defined as a set of non-overlapping regions of a genome, where each segment is assigned one of a small set of labels. Manually or automatically generated classifications, such as the examples above, are easily represented as segmentations, with a segment for each genomic locus. Labels may correspond to different types of functional elements — intron, exon, promoter — or to different subtypes of a single element — genes with high, medium or low expression. A user can then employ Segtools to explore how the labels relate to transcription factor binding sites, peaks of histone modification, or any other annotation. Note that some Segtools commands can operate on sets of regions that contain overlapping segments, such as overlapping transcripts. Throughout this manuscript and the Segtools documentation, we refer to this more relaxed form of segmentation as an *annotation*. Segtools generates results in tab-delimited text and image formats, and can summarize them in a single HTML report. Furthermore, Segtools analyses are easy to perform, script, and incorporate into existing analysis pipelines, making them useful for both manual and automated exploration.

## Implementation

Segtools is implemented as a collection of Python modules that process input files and output results in tab-delimited data files. These output files are then processed by visualization code written in R to generate plots.

### Usage

Segtools provides a set of tools for analyzing segmentations, a subset of which are summarized in Table 1. These tools can be run via a command-line or Python interface to create or compare segmentations and to visualize the properties of a segmentation and its relationships with provided annotations. A typical workflow involves 1) running the length-distribution, nucleotide-frequency, and transition commands on a segmentation to get a high-level view of its structure and the relationships among labels, 2) running signal-distribution against a set of signal tracks such as ChlP-seq signal intensities, and aggregation and overlap against a collection of annotations set such as genes, TSSs, enhancer sites, insulator sites, repetitive regions, CpG islands, or any other (potentially subcategorized) region sets, and then 3) generating an HTML report to collate these results. Because each Segtools command performs an independent analysis against a single annotation file, such a workflow is trivial to parallelize.

**Table 1:** Segtools analysis commands (segtools-...)

Command	Input	Output	Visualization
length-distribution	S	Segment length distribution by label	violin, bar plots (Figure 2)
nucleotide-frequency	S, G	Mono-/dinucleotide frequency by label	heat map (Figure IB)
signal-distribution	S, G	Signal mean and variance by label	heat map (Figure 3)
transition	S	Transition frequency between labels	heat map, graph diagram

aggregation	S, A	Label density around annotations	line plot (Figure 1A)
compare	S, S	Edit distances among all pairs of labels	heat map
overlap	S, A	Overlap of annotations by segments	PR curve, heat map

preprocess	A	An annotation in binary format	
flatten	S, A	Segmentation with a label for every combination of labels in the input segmentations	
feature-distance	S, A	Distance from each segment to nearest feature	
html-report	S	HTML summary of Segtools command outputs	

The commands offered by the Segtools package and their associated inputs and outputs. The first four commands analyze a single segmentation. The following three commands compare a segmentation to another segmentation or to an annotation. The last four commands generate no visualizations and are utilities to be used in conjunction with the other Segtools commands. In each row of the table, the second column indicates the input file types ("S" for segmentation, "A" for annotation, "G" for genomedata), and the fourth column indicates how the outputs are visualized (and a reference to an example figure in this article if one exists). "PR curve" refers to a precision-recall curve.

**Figure 1:**
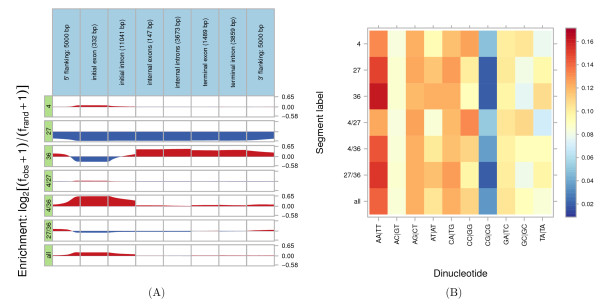
**Histone modifications**. Segtools plots for a segmentation of histone modification peaks produced with flatten (4: H3K4me3; 27: H3K27me3; 36: H3K36me3; 4/27: H3K4me3+H3K27me3; 4/36: H3K4me3+H3K36me3; 27/36: H3K27me3+H3K36me3; all: H3K4me3+H3K27me3+H3K36me3). **A**) The relative enrichment of these labels around active GENCODE release 3c protein-coding genes. "Manual" and "Auto" gene annotations from the UCSC Table Browser were merged and only protein-coding transcripts active in K562 (RPKM values in top 25%) were retained. Genes are split into idealized components: flanking regions; initial, internal, and terminal exons and introns, with the mean length of each component in parentheses. Enrichment is calculated as log_2 _(*f*_obs _+ 1)**/**(*f*_rand _+ 1), where *f*_obs _is the frequency at which the given label is observed at the given offset, and *f*_rand _is the frequency expected at random, given the relative abundance of each segment label. **B**) Mean dinucleotide frequencies across segments of each label.

**Figure 2:**
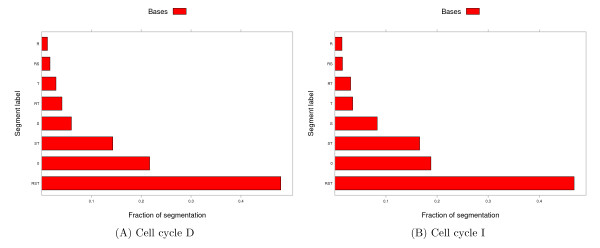
**Coverage of TSSs by gene expression label**. The figure plots, for two cell cycle experiments, the proportion of bases and segments that are covered by-each of the eight labels. The labels correspond to genes that are not expressed ("0"), expressed at a specific stage of the erythrocytic cycle ("R" for "ring", "T" for "trophozoite" and "S" for "schizont"), or expressed at multiple stages ("RS", "RT", "ST" and "RST"). Because each segment is of a fixed length (200 bp), the proportion of bases and segments covered is the same for each label.

**Figure 3:**
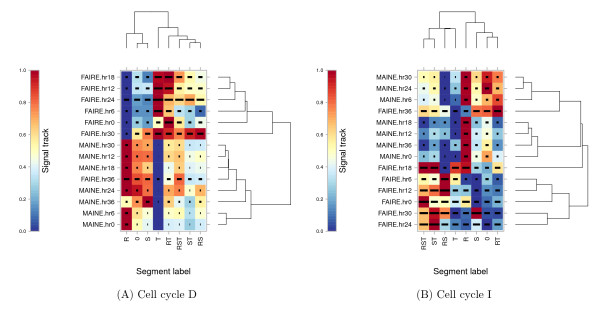
**MAINE and FAIRE signal at TSSs of genes segregated by time of expression**. Each panel plots the time series from the chromatin experiments versus the gene expression labels. The value in each cell, indicated by color, corresponds to the mean MAINE or FAIRE measurement around TSSs of genes with the given label. These values have been row-normalized to the range [0, 1]. The horizontal bar within each cell indicates the magnitude of the standard deviation, relative to all other cells. Panel (A) uses gene expression labels derived from the sorbitol-synchronized cell cycle, whereas panel (B) uses labels derived from the temperature cycling incubator synchronized cell cycle. In both panels, the vertical and horizontal axes of the heat maps shown have been ordered using hierarchical clustering.

As input, segmentations are accepted in Browser Extensible Data (BED) or General Feature Format (GFF) formats, with the "name" column used as the segment label. Point and region annotations are accepted in BED or GFF formats with the "name" column as an optional grouping variable, gene annotations in Gene Transfer Format (GTF), and signal annotations in Genomedata format [9]. As output, each command produces a tab-delimited text file containing the primary results, and most commands also produce a visualization of the results. A summary of the data outputted by each command is shown in Table 1, and the specific visualizations are as follows:

• The aggregation command produces a plot in which the x-axis is either a specific type of point annotation (such as a TSS) or a region (such as an exon), and the vertical axis is the relative enrichment of a given label at each position (see Figure 1A).

• The length-distribution command produces two visualizations: (1) a stacked collection of violin plots, each showing the distribution of segment lengths for one label, and (2) a bar plot showing the fraction of the segmentation (both in terms of bases and segments) that is occupied by each label (see Figure 2).

• The nucleotide-frequency command produces a heat map in which rows are segment labels, columns are dinucleotides, and values are frequencies of the given dinucleotide in the given label (see Figure 1B).

• The overlap command produces a series of precision-recall plots, one per annotation label. In each plot, every point corresponds to a segmentation label, the x-axis is the percentage of annotation labels that overlap the segmentation label, and the y-axis is the percentage of segmentation labels that overlap the annotation label. The command also produces a heat map in which rows are segment labels, columns are annotation groups, and values represent the fraction of overlap between segments of the given label and annotations of the given group. This overlap fraction is either in terms of segments or bases overlapped.

• The signal-distribution command produces a heat map in which rows are data tracks, columns are segment labels, and values represent the mean data value associated with the given label (see Figure 3).

• The transition command produces a heat map in which rows and columns are segment labels, and values represent the frequency with which the row label occurs immediately following the column label. The command also produces a graph visualization of the same data, in which nodes are labels, and edges represent transition frequencies. The command provides options to include only edges corresponding to high-frequency transitions.

Online documentation linked from the project web page contains complete usage information for each command.

## Results

### Case study 1: histone modifications in the human genome

Certain post-translational covalent modifications of histones are associated with gene expression [10-13], with specific combinations known to act cooperatively [14,15]. To demonstrate Segtools's functionality, we generated a segmentation from the ChlP-seq "peaks" (genomic loci exhibiting significantly elevated read count) for core histone H3 methylated at three different lysine residues (H3K4me3, H3K27me3, H3K36me3). The Broad Institute produced these data from the chronic myelogenous leukemia cell line K562 as part of the ENCODE Project [16], and we downloaded them from the UCSC Table Browser [17] on assembly NCBI36.

We compared the segmentation against GENCODE [18] version 3c gene annotations and transcription start sites (TSSs). We classified a gene as active when the number of ENCODE Project RNA-seq [19] reads per kilobase per million mapped reads (RPKM) in the gene exceeded the 75th percentile and as inactive when the gene had 0 RPKM. We classified TSS as active when it had at least 2 K562 cytosolic poly(A)^+ ^CAGE tags mapped from the ENCODE Project CAGE data [20], and as inactive when the TSS had 0 CAGE tags. First, we used flatten to create a segmentation in which the label for each segment corresponds to the combination of histone modifications with a peak at that segment. For example, the "4/27" label corresponds to regions spanned by both H3K4me3 and H3K27me3 ChlP-seq peaks. We then used aggregation in "gene mode" to visualize the enrichment of each label around the 11,693 protein-coding GENCODE genes active in the K562 cell line. Consistent with previous studies, Figure 1a shows the enrichment of H3K4me3 (4) around active transcription start sites in the first row, depletion of H3K27me3 (27) around active genes in the second row, and enrichment of H3K36me3 (36) in the bodies of actively-transcribed genes in the third row.

Then we created Figure 1b, with nucleotide-frequency. It shows the increased frequency of CpG in all labels that include promoter-associated H3K4me3 (4) peaks.

Finally, we used overlap to explore each label's predictive power for protein-coding TSS activity. With precision (also known as the positive predictive value) of 70.2% and and recall (or sensitivity) of 54.2%, segments high in both H3K4me3 and H3K36me3 were most predictive of overlapped TSSs being active. Surprisingly, segments high in all three histone modifications were the next most predictive of TSS activity, with precision of 68.7% and recall of 20.1%, suggesting that the presence of the other two histone modifications compensates for the inhibitory effect of H3K27me3. Segments with H3K27me3 alone were the most predictive of inactive TSSs, with precision of 95.2% and recall of 30.7%, though segments also high in H3K36me3 spanned an additional 5.2% of the inactive TSSs with a precision of 83.6%. In general, Segtools analyses are quick and parallelize easily. For this case study, the flatten analysis, which operated on three segmentations consisting of around 61,000 segments spanning ~50% of the human genome, required only 15 s on a single 2.33 GHz Intel Xeon CPU. The nucleotide-transition command processed the 1.6 billion bases spanned by the segmentation in 4 min, the overlap command summarized the intersection between these segments and 73,000 transcription start sites in 17 s, and the aggregation aggregated the segmentation over 9,000 gene models in 2 min.

### Case study 2: gene expression and local chromatin structure in the *Plasmodium falciparum *genome

We used Segtools to investigate the relationship between gene expression and local chromatin structure in *Plasmodium falciparum*, the parasite responsible for the most lethal form of malaria. Le Roch et al. [21] performed microarray expression assays in two time series across the *Plasmodium *erythrocytic cell cycle, corresponding to cell cycle synchronization performed with a 5% D-sorbitol treatment (cell cycle D) and a temperature cycling incubator (cell cycle I). Recently, these data were complemented with cell cycle time series data from two assays that measure local chromatin structure [22]: formaldehyde-assisted isolation of regulatory elements (FAIRE) [23] and MNase-assisted isolation of nucleosomal elements (MAINE) [24,25]. We used Segtools to investigate the extent to which the local chromatin profile varies as a function of gene expression.

Our analysis consisted of three steps. First, we identified genes that were significantly expressed in each of the three primary stages of the erythrocytic cycle: ring, trophozoite and schizont. To do so, we applied the statistical criterion from [21], and we required that the gene be expressed either in the "early" or "late" gene expression experiment for the given stage. This procedure was carried out separately for the two cell cycle data sets (D and I). Second, we used a previously curated set of transcription start sites (TSSs) [26] to identify genes with a single, known TSS, and then we labeled these TSSs with one of eight labels (R, S, T, RS, RT, ST, RST, 0) indicating the stages during which the gene is expressed. This labeling was accomplished by creating a BED file for each stage and then using flatten to merge the separate files into a single segmentation. The flattening was carried out separately for each cell cycle data set, resulting in two distinct labelings. Third, we applied several Segtools commands to each of the two segmentations, using a Genomedata archive that contained the FAIRE and MAINE data.

Figure 2 shows the results of applying length-distribution. Because we selected a 200 bp window around each TSS, the percent coverage by "Segments" or "Bases" is identical so we specified -no-segments to only plot the base coverage. The figure shows that a large proportion (47%-48%) of genes with known TSSs are expressed in all three stages of the erythrocytic cycle, and only a small proportion (10%-13%) are expressed, or at least accessible to transcription factors, exclusively in a single stage. This observation is consistent across the two cell cycles. Altogether, the data indicates that only a small proportion of the genes can be expressed in a stage specific manner.

Figure 3 shows the distribution of MAINE and FAIRE values over the course of the erythrocytic cell cycle as a function of different gene expression classes, produced using signal-distribution. Each cell corresponds to one expression label and one time point. The color of each cell indicates the strength of the MAINE or FAIRE signal in TSSs with the corresponding label. Each row of the plot is linearly scaled so that the minimum and maximum values are 0 and 1, respectively. Horizontal lines within the plot indicate the magnitude of the standard deviation in a given cell, relative to all other cells. Rows and columns have been ordered using the hierarchical clusterings shown on the top and right of each heat map. These two plots exhibit several intriguing features.

First, we note that the hierarchical clusterings shown along the right edge of both panels indicate that the FAIRE measurements at the end of the erythrocytic cycle (hr36) most closely resembles MAINE measurements (at hours 12, 18 and 30 in cell cycle D and hours 6, 24 and 30 in cell cycle I). This observation — that the FAIRE measurement of open chromatin at hr36 resembles measurements of closed chromatin — is consistent with the model proposed by Ponts et al., in which the parasite strongly compacts its chromatin in preparation for egress from the red blood cell at the end of the erythrocytic cycle. Second, we note that the genes expressed exclusively at the beginning of the cell cycle (R - ring stage) show an extremely strong and complementary pattern to genes expressed during the middle of the cell cycle (T - trophozoite stage). This pattern is particularly strong in cell cycle D (panel A), but also appears in cell cycle I (panel B). Apparently, ring-specific genes exhibit closed chromatin around their TSSs, whereas trophozoite-specific genes exhibit open chromatin around their TSSs. This pattern is consistent across nearly the entire cell cycle, with the possible exception of hr36, suggesting that local chromatin structure may contribute to stage-specific gene expression, but that local chromatin dynamics may not be the only mechanism regulating gene expression.

Overall, the figure shows relatively little correlation between the time at which a gene is expressed and changes in local chromatin structure. Canonically, time points 0, 6 and 12 of the MAINE/FAIRE data correspond to the ring stage, time points 18 and 24 correspond to the trophozoite, and time points 30 and 36 correspond to schizont. The absence of a strong correlation between time of expression and the degree of local chromatin compaction suggests that, though Ponts et al. have clearly demonstrated that local chromatin structure changes over the course of the erythrocytic cycle, the current analysis does not support a model in which the degree of chromatin compaction around the TSS directly correlates with the expression of the gene. Apparently, a more complex model that integrates additional types of data, such as transcription factor binding and histone modification profiles, is required to fully understand *Plasmodium's *unusual gene expression machinery.

## Discussion and Conclusions

Segtools enables the rapid exploration of a bird's-eye view of complex multi-label data, allowing researchers to easily generate and confirm hypotheses.

One challenge in creating any software toolkit is to define the scope of the project, treading a line between solving many problems and solving a few problems well. Segtools is specifically targeted toward the analysis of segmentations, which we believe will become an increasingly prevalent and useful way to make sense of collections of parallel genomic data sets. Segtools emphasizes the efficient calculation of summary statistics and publication quality visualizations thereof. Indeed, all of the figures in this article were generated directly by Segtools with no subsequent processing. Statistical hypothesis testing — that is, testing for the enrichment of a specific annotation label within a specific segment label — has not been implemented, primarily because many such tests have been proposed [27-30]. Full implementations of such tests are available in R, and Segtools can interoperate seamlessly with these existing functions. Similarly, we have not attempted to build into Segtools sophisticated functionality for pre-filtering segmentations and annotations. Segtools currently includes limited pre-processing functionality, in the form of the flatten and feature-distance commands; however, for sophisticated logical filtering operations, a toolkit such as Galaxy [31] or BEDtools [7] should be used prior to Segtools analysis.

Given the growing availability of large-scale heterogeneous functional genomics data sets, methods that allow us to quickly and easily summarize and make sense of these data are in growing demand. The two case studies included in this paper demonstrate how one can use Segtools to pick out interesting results from complex data. Individually examining many potential hypotheses one-by-one would prove laborious and difficult, but Segtools makes it trivial to perform a broad battery of exploratory data analyses and find the important features of segmentation results.

## Availability and requirements

**Project name: **Segtools

**Project home page: **http://noble.gs.washington.edu/proj/segtools

**Operating systems: **Linux, Mac OS X

**Programming language: **Python 2.5.1-2.7, R ≥ 2.10

**Other requirements: **Segtools requires NumPy ≥ 1.3, two R packages (latticeExtra, reshape), one Python package (RPy2 ≥ 2.1.3), and the Genomedata Python package for the two commands that process Genomedata files. Segtools can then be easily installed by typing easy_setup segtools at the shell prompt. Segtools can also be acquired using our installation script that attempts to install Segtools and all missing dependencies, or it can be downloaded as a virtual machine complete with all dependencies. See the project home page for additional installation instructions.

**License: **GNU GPL

**Any restrictions to use by non-academics: **none

## Authors' contributions

MMH and WSN conceived of and supervised the project. OJB and MMH developed the software. OJB and WSN carried out the experiments. OJB, MMH, NP, KGLR and WSN wrote the manuscript. All authors read and approved the final manuscript.
